# Polymorphism of Bismuth Sesquioxide. II. Effect of Oxide Additions on the Polymorphism of Bi_2_O_3_

**DOI:** 10.6028/jres.068A.020

**Published:** 1964-04-01

**Authors:** Ernest M. Levin, Robert S. Roth

## Abstract

The effect of small oxide additions on the polymorphism of Bi_2_O_3_ was studied by means of high-temperature x-ray diffractometry. Solidus and occasional liquidus temperatures were approximated, so that tentative partial phase diagrams for 33 oxide additions were constructed. Only the monoclinic and the cubic forms of Bi_2_O3 were found to be stable. Other phases, frequently reported by previous investigators, such as tetragonal and body-centered cubic (b.c.c.), were shown to form metastably from cooled liquid or cubic. An impure b.c.c. phase of distinct but variable composition appeared in systems of ZnO, PbO, B_2_O_3_, Al_2_O_3_, Ga_2_O_3_, Fe_2_O_3_, SiO_2_, GeO_2_, TiO_2_, and P_2_O_5_. The impure b.c.c. phase in the systems with SiO_2_, GeO_2_, and TiO_2_ melted congruently about 100 °C above the m.p. of Bi_2_O_3_. The impure b.c.c. phase was formed metastably in systems with Rb_2_O, NiO, MnO, CdO, V_2_O_5_, and Nb_2_O_5_; the conditions of formation were dependent on composition, preparation, and heating schedules. The impure b.c.c. phases, both stable and metastable, had smaller unit cell dimensions than that of pure Bi_2_O_3_.

## 1. Introduction

Part I of this paper was an attempt to clarify the stable and metastable relationships of pure Bi_2_O_3_. It can be seen in table 1 of that part, however, that I several investigators [1,2,3]^1^ have reported phases of Bi_2_O_3_ which contained, or were contaminated by, other oxides. Sillén [1] obtained a body centered cubic (b.c.c.) form by fusing Bi_2_O_3_ in porcelain, or with A1_2_O_3_ or Fe_2_O_3_, for 5 min at 900 °C. He suggested the unit cell formula Me_2_Bi_24_O_40_. Fusion of Bi_2_O_3_ in a porcelain crucible for 20 min yielded cubic Bi_2_O_3_. Schumb and Rittner [2] also obtained the impure b.c.c. phase by fusing Bi_2_O_3_ at 875 °C in porcelain or with SiO_2_. By quenching the fused mixture in water, they produced an impure cubic (C) phase. Gattow and Schröder [3] reported impurity forms of b.c.c., C, and tetragonal-symmetry, designated respectively, as γ*, δ*, β*.

The previous work can be questioned on two grounds: Firstly, in most cases the exact compositions were not controlled and were not known; secondly, in no instances were the phases studied at temperature and, consequently, might not represent stable equilibrium phases. Quenched liquids, for example, could hardly be expected to give equilibrium phases for any temperature. Furthermore, as the stable phases of Bi_2_O_3_ have been shown to be monoclinic and cubic-bismuth oxide, any other phase, e.g., impure tetragonal (Tet) or b.c.c. must represent either a metastable form of Bi_2_O_3_ or a discrete phase, whose composition limits does not include Bi_2_O_3_.

The major objective of this portion of the study, therefore, was to obtain information on the impure forms of bismuth oxide, in particular, on the b.c.c. phase. To eliminate objections applicable to previous work, mixtures were formulated from pure materials and of known compositions and were studied in a high-temperature x-ray diffractometer furnace.

## 2. Materials and Methods

### 2.1. Materials

For the admixture study, reagent grade chemicals (ACS) or those of higher purity were used. Starting materials for formulating oxide mixtures with Bi_2_O_3_ were as follows: Li_2_CO_3_, Rb_2_CO_3_, NiO, ZnO, CdO, MgO, CaCO_3_, SrCO_3_, BaCO_3_, PbO, B_2_O_3_, Al_2_O_3_, Ga_2_O_3_, Fe_2_O_3_, MnCO_3_, Sb_2_O_3_, Lu_2_O_3_, Sm_2_O_3_, La_2_O_3_, SiO_2_, GeO_2_, TiO_2_, TeO_2_, SnO_2_, ZrO_2_, CeO_2_, P_2_O_5_, V_2_O_5_, Sb_2_O_5_, Ta_2_O_s_, Nb_2_O_5_, Cr_2_O_3_, WO_3_, and MoO_3_. These materials were chosen from crystal chemistry considerations, such as charge, ionic radius, coordination number, and polarizability of the cations.

### 2.2. Preparation of Mixtures

Binary mixtures were formulated from Bi_2_O_3_ and a second substance, to give an atomic ratio in most cases of 12Bi to 1Me, where Me represents the second cation. The intent was to determine which oxides formed Sillén’s [1] impure b.c.c. phase at the above ratio. Thus for monovalent, trivalent, and pentavalent cations, oxide compositions would correspond to 12Bi_2_O_3_:Me_2_O, 12Bi_2_O_3_:Me_2_O_3_, and 12Bi_2_O_3_:Me_2_O_5_. For divalent, tetravalent, and hexavalent cations, oxide compositions would correspond to 6Bi_2_O_3_:MeO, 6Bi_2_O_3_:MeO_2_, and 6Bi_2_O_3_: MeO_3_. Additional compositions were prepared in many of the systems, as designated on the individual phase diagrams.

Preliminary treatment of the mixtures consisted of three cycles of grinding together calculated amounts of the starting materials, pressing the material in a mold, and then heating the disk at a temperature below the solidus, as described in previous publications [4,5].

### 2.3. Apparatus

The high-temperature, x-ray diffractometer furnace noted in Part I was used also for the admixture study. The modification of the sample holder [6] which permitted the use of a thin layer of powered specimen was of especial importance for this part of the study. X-ray diffraction patterns of crystalline phases could be obtained in the presence of liquid, which did not flow off the platinum holder. Thus, it was possible to approximate solidus temperatures (±10°) and under favorable conditions even the liquidus temperatures.

## 3. Results and Discussion

### 3.1. Phase Diagrams

#### a. General Remarks

**Figure 1:**
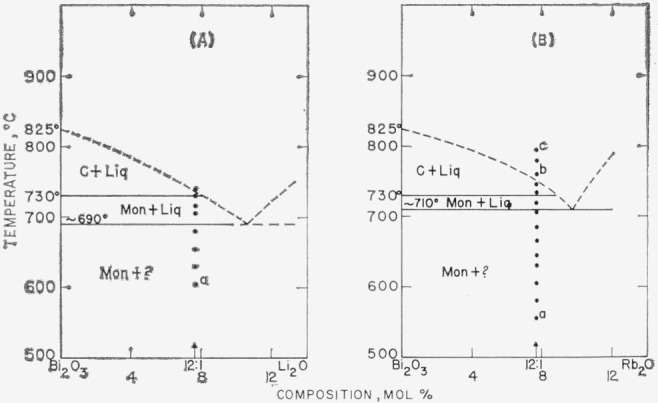
*Bi_2_O_3_* rich regions of *Bi_2_O_3_–R_2_O* systems, as determined from high-temperature x-ray diffraction data Phases: Mon—monoclinic, C—cubic, ?—unknown, Liq—liquid (A) Bi_2_O_3_–Li_2_O a—Trace of unknown phase observed. (B) Bi_2_O_3_–Rb_2_O a—No second phase seen, b—Rb_2_O apparently volatilizes from liquid, c—Liquid cools to b.c.c. phase: a=10.22A.

The data obtained by use of the high-temperature x-ray furnace can be presented in the form of phase diagrams, as shown in figures 1 through 6. Arrangement of the figures is according to the Periodic Table. Within each figure, diagrams are arranged, in general, according to a combination of the Periodic Table subgroups and ionic radii of the atoms. As no table of data is given, figure captions include selected notes. In most instances, the rate of disappearance of old phases and of the appearance of new ones on the indicated boundary curves was not rapid but took place over a temperature interval. Phases which have been interpreted as nonequilibrium ones are not shown in the diagrams.

It is emphasized that these phase diagrams represent the best interpretation of the data within the limitations of the experimental method. The major limitation is due to volatility of the samples or of the constituents. The high surface-to-volume ratio inherent with a thin film method, increases the effect of volatilization on composition. Thus, verification of equilibrium by long soaking periods, especially at high temperatures, was precluded.

#### b. General Conclusions

**Figure 2:**
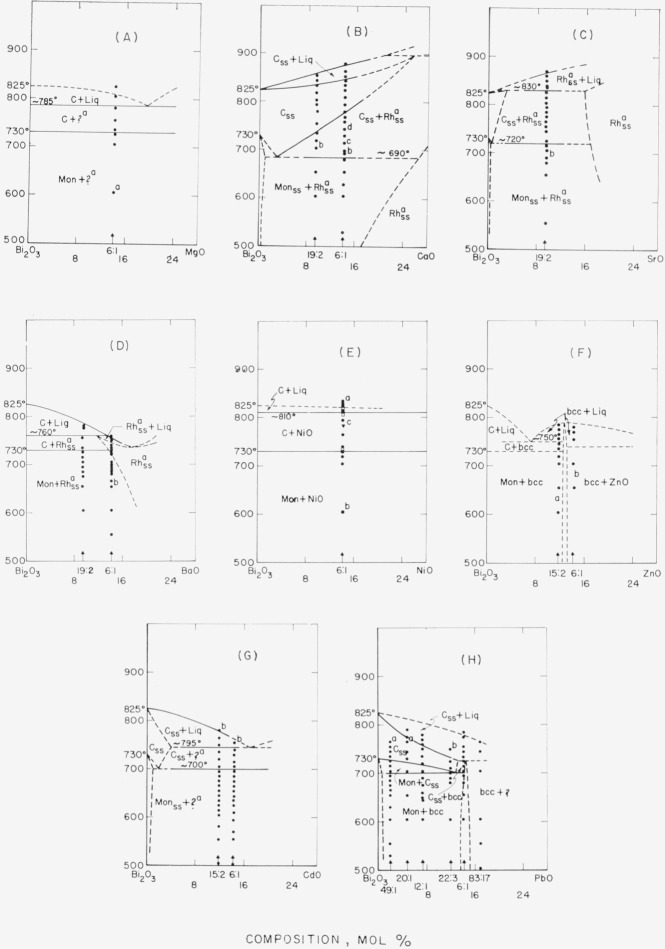
*Bi_2_O_3_* rich regions of *Bi_2_O_3_–RO* systems, as determined from high-temperature x-ray diffraction data Phases: Mon—monoclinic, C—cubic, b.c.c.—body-centered cubic, ?—unknown, Rh—rhombohedral, ss—solid solution, Liq—liquid (A) Bi_2_O_3_–MgO a—Second phase, possibly MgO. (B) Bi_2_O_3_–CaO a—Described by Aurivillius (1943) [7]. b—Cubic Bi_2_O_3_ ss starting to form, c—Cubic Bi_2_O_3_ ss increasing; Mon Bi_2_O_3_ decreasing, d—Mon Bi_2_O_3_ gone. The 6:1 composition slow-cooled from 700 °C (after 107 hr soaking period) shows single phase Rh_ss_. See table 1 for unit cell dimensions. (C) Bi_2_O_3_–SrO a—Described by Sillén and Aurivillius (1939) [8]. b—Rh_ss_ increasing; Mon decreasing. See table 1 for unit cell dimensions. (D) Bi_2_O_3_–BaO a—Described by Aurivillius (1943) [7]. b—Rh_ss_ increasing; Mon decreasing. See table 1 for unit cell dimensions. (E) Bi_2_O_3_—NiO a—Liq cools to b.c.c. ●—First heat □—b.c.c. reheated ■—Superposition of ● and □. ▼ -Reheated sample cooled from liq to 780 °C b—b.c.c. decomposing to Mon. c—Cubic phase when cooled to room temperature shows b.c.c. (F) Bi_2_O_3_—ZnO a—No Mon detected. b—ZnO detected only in specimen soaked 107 hr at 700 °C and then slow-cooled. The two compositions studied indicate that the b.c.c. phase is stable and melts congruently. Beyond this the diagram is conjectural. G) Bi_2_O_3_—CdO a—Possibly one of Sillén’s reported CdO—Bi_2_O_3_ phases [9]. b—Liq cools to b.c.c. (H) Bi_2_O_3_—PbO a—C_ss_ cools to b.c.c. b—C_ss_+ Liq cools to b.c.c.

**Figure 3:**
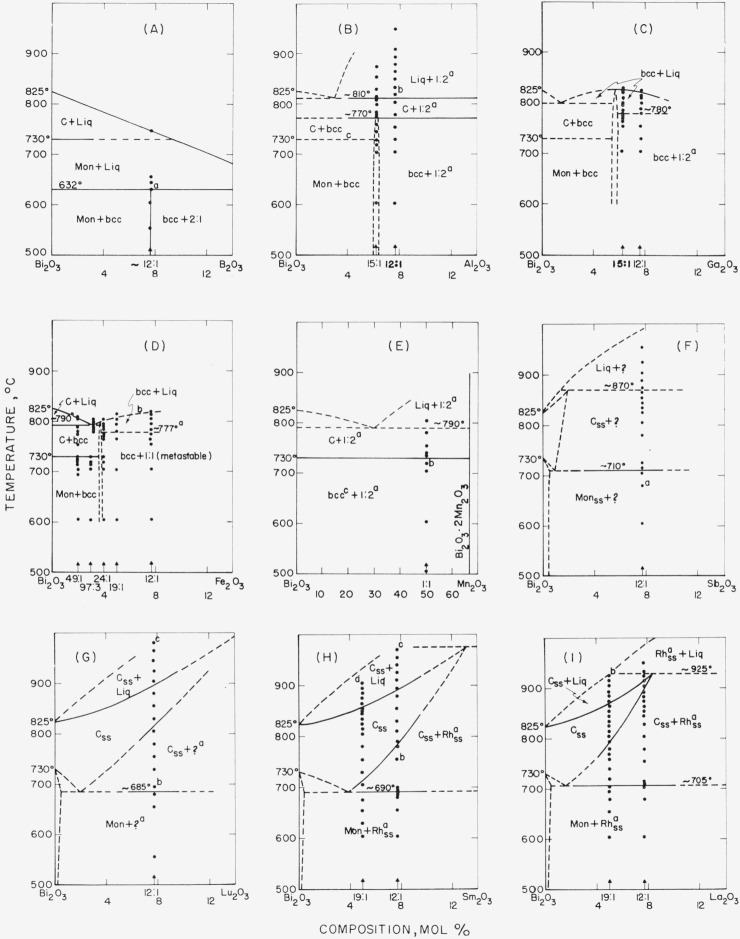
*Bi_2_O_3_* rich regions of *Bi_2_O_3_*–*R_2_O_3_* systems, as determined from high-temperature x-ray diffraction data Phases: Mon—inonoclinic, C—cubic, b.c.c.—body-centered cubic, ?—unknown, Rh—rhombohedral, ss—solid solution, Liq—liquid. (A) Bi_2_O_3_—B_2_O_3_ Diagram taken from Levin et al. [4]. a—Unreacted 2Bi_2_O_3_·B_2_O_3_(2:1) and Mon Bi_2_O_3_ at lower temperatures form single phase b.c.c. B) Bi_2_O_3_—Al_2_O_3_ a—Isostructural with 1:2 compounds in systems of Bi_2_O_3_ with Fe_2_O_3_, Ga_2_O_3_, and Mn_2_O_3_, according to unpublished work of R. S. Roth. b—Very strong peak at *d* value about 2.9A; probably orientation of Bi_2_O_3_·2Al_2_O_3_. c—Effect of Al_2_O_3_ on Mon to C transition temperature not determined. (C) Bi_2_O_3_—Ga_2_O_3_ a—Isostructural with 1:2 compounds in systems of Bi_2_O_3_ with Fe_2_O_3_, Al_2_O_3_, Mn_2_O_3_, according to unpublished work of R. S. Roth. The two compositions studied indicate that the b.c.c. phase is stable and melts congruently. Beyond this the diagram is conjectural. (D) Bi_2_O_3_—Fe_2_O_3_ (Does not obey phase rule.) a—May represent metastable eutectic between b.c.c. and metastable BiFeO_3_. b—Liquidus curve does not show maximum temperature at melting of b.c.c. phase. The BiFeO_3_ phase is believed to be metastable because the compound composition can never be made single phase, according to the unpublished work of R. S. Roth. (E) Bi_2_O_3_—Mn_2_O_3_ (Note different composition scale.) MnCO_3_ used as starting material, and mixture ground in alcohol, which tends to give b.c.c.[17]. a—Isostructural with 1:2 compounds in systems of Bi_2_O_3_ with Fe_2_O_3_, Al_2_O_3_, Ga_2_O_3_, according to unpublished work of R. S. Roth, b—Samples at this temperature and below show b.c.c., believed to be metastable, as it disappears at Mon to G inversion (730 °C). c—Mon Bi_2_O_3_ probably stable phase (see b). (F) Bi_2_O_3_—Sb_2_O_3_ a—Above about 650 °C oxidation state of the antimony is unknown. Starting with Sb_2_O_5_, same phases occur, but solidus appears to be lower (~855 °C). (G) Bi_2_O_3_—Lu_2_O_3_ a—Second phase observed, with major *d* value at 2.95A. b—As Mon transorms to C, unreacted Lu_2_O_3_ transforms to ? phase. c—Liq+C_ss_ cools to metastable tetragonal_ss_. (H) Bi_2_O_3_—Sm_2_O_3_ a—Essentially isostructural with Rh_ss_ phase in CaO—, SrO—, and BaO — Bi_2_O_3_ systems. See table 1 for unit cell dimensions. b—When cooled to 450 °C shows Rh_ss_+ tetragonal (Tet)_ss_ (metastable). c—Liq+C_ss_ cools to Tet_ss_+C_ss_ (both metastable). d—Liq+C_ss_ cools to Tet_ss_ (metastable). Single phase Tet_ss_ (19:1) reheated to 625 °C transforms first to C_ss_+Rh_ss_, then the C_ss_ transforms to the equilibrium Mon phase. (I) Bi_2_O_3_—La_2_O_3_ a—Essentially isostructural with Rh_ss_ phase in CaO—, SrO —, and BaO — Bi_2_O_3_ systems. See table 1 for unit cell dimensions. b—Cools to C_ss_ (metastable)

**Figure 4:**
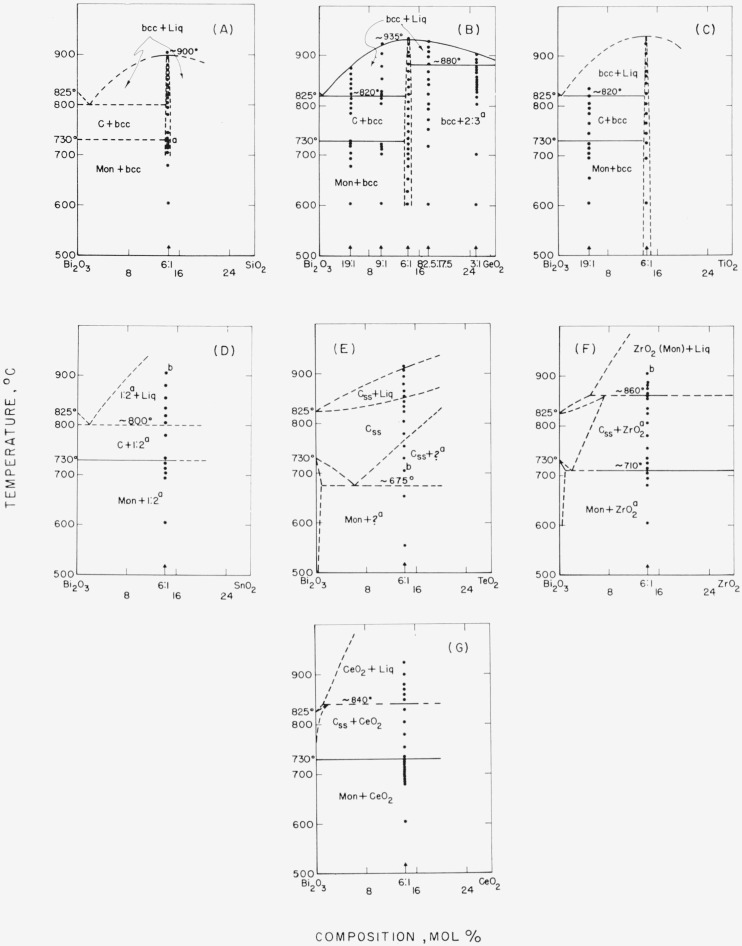
*Bi_2_O_3_* rich regions of *Bi_2_O_3_ —RO_2_* systems, as determined from high-temperature x-ray diffraction data Phases: Mon—monoclinic, C—cubic, b.c.c.—body-centered cubic, ?—unknown, ss—solid solution, Liq—liquid (A) Bi_2_O_3_-SiO_2_ a—Unreacted Mon transforms to C, on heating. Composition studied shows that the b.c.c. phase (6:1) melts congruently. Beyond this the diagram is conjectural. (B) Bi_2_O_3_–GeO_2_ a—Unpublished work of C. R. Robbins, NBS. Compositions studied show that the b.c.c. phase melts congruently, near to but not necessarily at the 6:1 composition. (C) Bi_2_O_3_—TiO_2_ Compositions studied show that the b.c.c. phase melts congruently, near to but not necessarily at the 6:1 composition. (D)Bi_2_O_3_—SnO_2_ a—Compound described by R. S. Roth [14]. b—1:2 + Liq cools to 1:2 + Mon + b.c.c. (trace). (E) Bi_2_O_3_–TeO_2_ Oxidation state of tellurium at high temperature is unknown. a—Unknown composition, apparently of cubic symmetry. b—Unit cell dimensions at 700 °C: C_ss_, a=5.63A; ?, a=5.57A (F) Bi_2_O_3_—ZrO_2_a—ZrO_2_ does not show in x-ray pattern below solidus. b—Liq + ZrO_2_ (Mon) cools to Tet Bi_2_O_3ss_. No b.c.c. found in this system although reported by Aurivillius and Sillén [15]. (G) Bi_2_O_3_—CeO_2_. No b.c.c. found in this system although reported by Aurivillius and Sillén [15].

**Figure 5:**
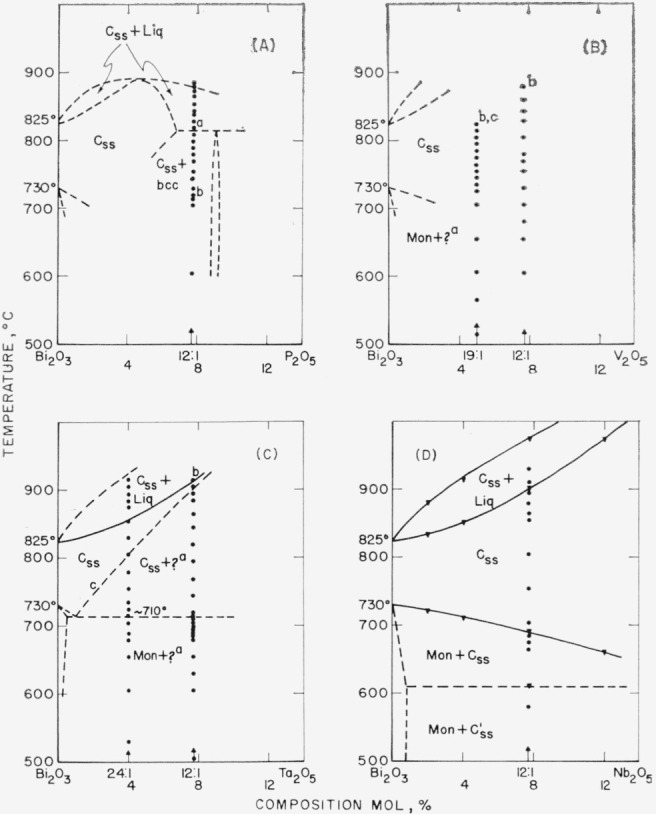
*Bi_2_O_3_* rich regions of *Bi_2_O_3_—R_2_O_5_* systems, as determined from high-temperature x-ray diffraction data Phases: Mon—monclinic, C—cubic, C′—pseudocubic, b.c.c.—body-centered cubic, ?—unknown, ss—solid solution, Liq—liquid (A) Bi_2_O_3_–P_2_O_5_ a—The b.c.c. phase decreases markedly in amount at about 815 °C, but persists in diminishing amounts to about 850 °C. It is possible, therefore, that the true solidus is at 850 °C and (1) a 2-phase region of C_ss_ + ? exists between 815 ° and 850 °C or (2) the decomposition temp, of b.c.c. should be raised to 850 °C. b—The Mon to C transition with the high-temp, x ray occurs at about 720 °C. However, a sample held at 700 °C for 107 hr and slow-cooled does not show Mon. Therefore, the temperature of the Mon to C inversion is in doubt. (B) Bi_2_O_3_—V_2_O_5_ A more complete phase diagram could not be postulated because of inconsistencies in the temperatures of transitions and the relative amounts of phases present. a—b.c.c., if a stable phase; otherwise, unknown phase. b—Above 815 °, only C_ss_; between 715° and 815 °C, C_ss_ + b.c.c.; below 715 °C, phases present depend on previous heat treatment. Stability and approximate composition of b.c.c. could not be ascertained, c—C_ss_ cools to b.c.c. (C) Bi_2_O_3_–Ta_2_O_5_ a—Apparently cubic symmetry; composition may correspond to C′ phase in Bi_2_O_3_—Nb_2_O_5_ system [17] (about 4:1). b—Liq + C_ss_ cooled slowly shows only C_ss_ at all temps. c—Because of nonreversibility (see b), this boundary may actually be much lower, as in the Bi_2_O_3_—Nb_2_O_5_ system (D). (D) Bi_2_O_3_–Nb_2_O_5_ ▼—Values taken from phase diagram of Roth and Waring [17].

From inspection of all of the diagrams, several general conclusions become evident:

1. The only stable phases of pure bismuth oxide are Mon and C. The b.c.c. or Tet phases of pure Bi_2_O_3_ do not appear. The conclusion is consistent with the stability relationships for Bi_2_O_3_ as deduced in Part I.
2. Monoclinic Bi_2_O_3_ shows little or no solid solution.
3. The C phase of Bi_2_O_3_, however, may show extensive solid solution: and in such cases, the Mon to C transition temperature is lowered.
4. Except for the CdO and PbO systems (fig. 2 G& H), the effect of solid solution in the C phase is to raise solidus and liquidus temperatures.
5. A b.c.c. phase distinct from that of pure Bi_2_O_3_ appears in a number of systems. This b.c.c. phase may vary in composition for different systems (see figs. 2F, 3B & C, 4A, 5A), may melt congruently (fig. 4A, B & C), or melt incongruently (figs. 2H, 3A & B), and when stable is separated from Bi_2_O_3_ by a two-phase region.

#### c. Individual Phase Diagrams

In the following section, individual figures and selected diagrams will be discussed. Only two alkali oxide systems, representing extremes in cation radii, were studied (fig. 1). Both Li_2_O and Rb_2_O, in the region studied, were simple eutectic types with no solid solutions. It should not be inferred, however, that oxides of the intermediate cations necessarily would behave similarly.

For oxides of the divalent cations (fig. 2) the phase diagrams showed a number of variations, e.g., simple eutectic system (A), congruently melting b.c.c. phase (F), incongruently melting b.c.c. phase (H), C solid solution with liquidus and solidus raised (B), and C solid solution with liquidus and solidus lowered (H).

In the systems with CaO, SrO, and BaO, the rhombohedral solid solution phase described previously [7, 8] was found to be an equilibrium phase. Sillén and Aurivillius had found the phase in samples cooled rapidly from the liquid. The unit cell dimensions at 700 °C for the bismuth oxide-rich compositions of the solid solution phase are given in table 1.

Sillén and Sillén [9] report finding several phases in the Bi_2_O_3_-CdO system, one of which might be the unknown phase in the present study of the cadmium oxide system (fig. 2G).

The PbO-Bi_2_O_3_ system (fig. 2H) is interesting for several reasons. It varies significantly from the reported phase diagram of Belladen [10, 11]. The latter shows neither the Mon to C transition, nor the solid solution of PbO in Bi_2_O_3_, nor the b.c.c. phase at the 6Bi_2_O_3_·PbO composition. It is evident from the present diagram that the molal heat of fusion of Bi_2_O_3_ calculated by Kelley [12] from the liquidus curve of Belladen’s diagram (6800 cal/mole) is in error. The molal heat of fusion of Bi_2_O_3_ was discussed by Levin and McDaniel [4].

With regard to the systems of bismuth oxide with oxides of the trivalent cations, the Bi_2_O_3_-B_2_O_3_ system (fig. 3A) was reported previously [4], as determined by the quenching technique. However, one composition, 12Bi_2_O_3_:B_2_O_3_, was used to compare both methods and to show that agreement was satisfactory.

Apparently isostructural 1:2 compounds were found in systems of Bi_2_O_3_ with Al_2_O_3_ (fig. 3B), Ga_2_O_3_ (fig. 3C), and Mn_2_O_3_ (fig. 3E). An isostructural 1:2 compound also exists in the Bi_2_O_3_–Fe_2_O_3_ system; however, the high-temperature x-ray study revealed only a 1:1 compound (fig. 3D). The BiFeO_3_ phase is believed to be metastable, because according to the unpublished work of R. S. Roth, the compound composition can never be made single phase. It should also be noted that the portion of the diagram to the right of the b.c.c. phase does not obey the phase rule. Royen and Swars [13] who also have studied this system reported two Tet phases of approximately 30:1 and (12–13):1 compositions, a b.c.c. phase of composition 15:1, as well as 2:1 and 1:1 compounds. The present work does not substantiate the 30:1, (12–13) :1, and 2:1 compounds; and the composition of the b.c.c. phase appeared slightly greater than 24Bi_2_O_3_ :Fe_2_O_3_. As Royen and Swars obtained their compounds from fused mixtures, it is believed that some of them represented metastable states.

The system with the rare earth oxides Sm_2_O_3_ (fig. 3H) and La_2_O_3_ (fig. 3I), showed the same rhombohedral solid solution phase as was found in the CaO, SrO, and BaO systems (fig. 2B–D). The unit cell dimensions are given in table 1. The phase did not form in the Lu_2_O_3_ system (fig. 3G) nor in the MgO system (fig. 2A); consequently, the minimum cationic radius required for formation of this phase lies between that of Lu^3+^ and Ca^2+^ or between 0.85 and 0.99A (Ahrens).

In the systems studied with the group IV cations (fig. 4), SiO_2_ (A), GeO_2_ (B), and TiO_2_ (C) showed a congruently melting b.c.c. phase at or near the 6:1 composition. The phase diagrams provide positive proof, for the first time, that the impure b.c.c. phase is a discrete composition and not a solid solution phase of Bi_2_O_3_.

The Bi_2_O_3_-SnO_2_ system (fig. 4D) contains a 1:2 compound described by R. S. Roth [14] as having a distorted pyrochlore-type structure.

No b.c.c. phase was found in the ZrO_2_ and CeO_2_ systems, although this phase was obtained from fused mixtures by Aurivillius and Sillén [15]. As will be discussed later under the b.c.c. phase, cooling of the liquid or C phases tends to form metastable phases.

In the group V cations (fig. 5), systems with P_2_O_5_ (A) and V_2_O_5_ (B) showed inconsistencies which could not be reconciled, and these diagrams are most questionable. The systems with Ta_2_O_5_ (C) and Nb_2_O_5_ (D) are similar, and it is possible that the boundary curve in the Ta_2_O_5_ system between (Css+ ?) and Css should descend continuously as in the Nb_2_O_5_ system (see b and c in legend to fig. 5C).

**Figure 6:**
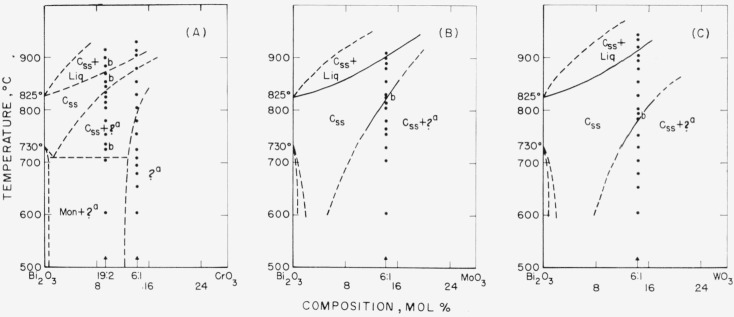
*Bi_2_O_3_* rich regions of *Bi_2_O_3_—RO_3_* systems, as determined from hiqh-temperature x-ray diffraction data Phases: Mon—monoclinic, C—cubic, ss—solid solution, ?—unknown, Liq— liquid (A) Bi_2_O_3_—CrO_3_ Compositions formulated from Cr_2_O_3_. Diagram shown on CrO_3_ basis because on calcining, specimens changed in color from green to red, and ? phase is similar to that formed with the hexavalent ions. a—Unknown composition, apparently pseudotetragonal (similar to the phase in the Bi_2_O_3_—MoO_3_ and Bi_2_O_3_—WO_3_ systems). b—Phases found at these temps, do not agree entirely with postulated phase boundaries, because system may be pseudobinary. (B) Bi_2_O_3_—MoO_3_ a—Unknown composition, apparently pseudotetragonal (similar to the phase in the Bi_2_O_3_—CrO_3_ and Bi_2_O_3_—WO_3_ systems). b—Reversibility demonstrated with high-temp, x ray. (C) Bi_2_O_3_—WO_3_ a—Unknown composition, apparently pseudotetragonal (similar to the phase in the Bi_2_O_3_—CrO_3_ and Bi_2_O_3_—MoO_3_ systems). b—Reversibility demonstrated with high-temp, x ray.

Finally, in the Bi_2_O_3_–RO_3_ systems (fig. 6), the three systems studied are similar, including the occurrence of a phase of apparently pseudotetragonal symmetry but of unknown composition. Gattow [16] prepared a mixed oxide 2Bi_2_O_3_·MoO_3_, by precipitation from solution, and studied it by means of x-ray and thermal analysis. Because of insufficient data, however, it was not possible to compare the unknown phase in the present study with Gattow’s.

### 3.2. Metastable Phases

The results of the high-temperature x-ray experiments were especially informative regarding the occurrence of metastable phases observed at room temperature in samples cooled from higher temperatures. Such information is included in the figure captions. Illustrative examples are as follows:

1. Liquid cooled to a metastable b.c.c. phase in Bi_2_O_3_ systems with Rb_2_O (see c to caption of fig. IB), NiO (a of fig. 2E), and CdO (b of fig. 2G).
2. Cubic or cubic solid solution cooled to a metastable b.c.c. phase in systems with NiO (c of fig. 2E) and V_2_O_5_ (c of fig. 5B).
3. Liquid+cubic solid solution cooled to metastable cubic solid solution in systems with La_2_O_3_ (b of fig. 3I), Ta_2_O_5_ (b of fig. 5C), and Nb_2_O_5_ [17].
4. Liquid+cubic solid solution also cooled to metastable Tet in systems with Lu_2_O_3_ (c of fig. 3G), Sm_2_O_3_ (b and d of fig. 3H), and Nb_2_O_5_ [17].
5. In the zirconia system, liquid+ZrO_2_ cooled to metastable tetragonal.

It is emphasized that an exhaustive study of the formation of metastable phases was not attempted. In general, only one cooling cycle for a limited number of compositions was studied in each system. It is apparent, however, from the frequency and diversity of the matastable phases found, that phases obtained by the cooling of fused mixtures or of high-temperature forms may well represent nonequilibrium states at all temperatures. It is not surprising, therefore, that previous investigators [1, 2, 13, 15] studying fused samples of unknown compositions obtained various impurity phases. Many of these are metastable phases and have no place in the equilibrium diagrams.

### 3.3. Body-Centered Cubic Phase

Table 2 gives the unit cell dimensions for the stable and metastable b.c.c. phases of bismuth oxide found in this study. The unit cell dimensions were obtained at room temperature; and except for Rb_2_O and Bi_2_O_3_ (see footnotes d and e, respectively) the samples were heated in sealed platinum tubes according to the schedule given in the column under “Final Heat Treatment”. As observed from the x-ray diffraction patterns, most of the compositions studied were not single phase but showed a second phase.

**Figure 7:**
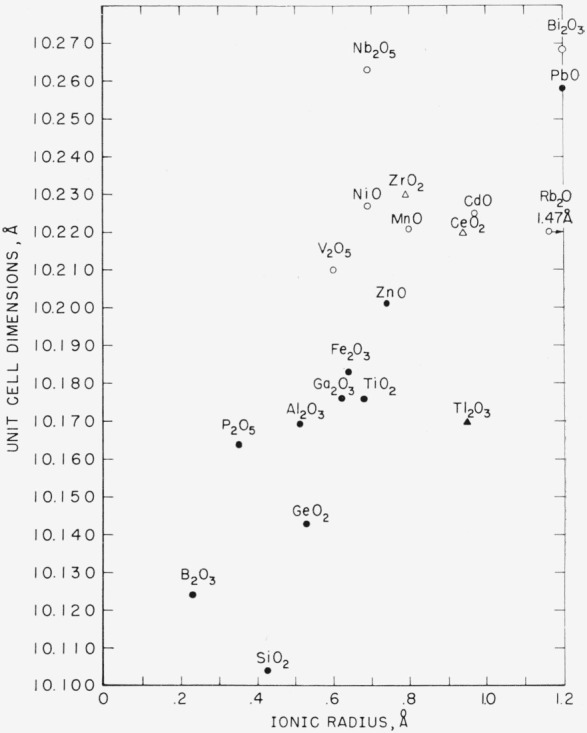
Unit cell dimensions versus ionic radius for impure body-centered cubic phases of *Bi_2_O_3_* Oxides shown in the figure refer to the impurity additions. See figures 2 through 5 for approximate compositions of the phases. ●—equilibrium phases ○—metastable phases ▲, △—unit cell dimensions after Aurivillius and Sillén [15].

Concerning the systems with a stable b.c.c. phase, it is seen that an exact 12Bi to 1Me atom ratio was not substantiated in most cases. Except for PbO and B_2_O_3_, systems with divalent and trivalent cations would show single phase b.c.c. at ratios of Bi to Me greater than 12:1. Systems with the tetravalent ions Si^4+^, Ge^4+^, and Ti^4+^ approached most closely the ideal ratio of 12Bi: 1Me, proposed by Sillén [1]. This conclusion is more apparent from figure 4A,B,C, where the b.c.c. phase in these systems is seen to melt congruently at a temperature about 100 °C above the melting point of Bi_2_O_3_. With the oxide of the pentavalent cation, P^5+^, the single phase b.c.c. composition appears to be less than 12Bi:1Me (fig. 5A). It can be seen from table 2 that within each valence group increased ionic radius of the cation is associated with increased unit cell dimensions of the b.c.c. phase. Unit cell dimensions versus ionic radius for all of the cations are plotted in figure 7. The general correlation between the two for the stable phases (solid points) is seen to be good, although not linear.

An interesting and surprising finding is that oxides of cations so diverse in ionic radius, oxygen coordination number, and polarizability as Zn^2+^, B^3+^, Ti^4+^, and Pb^2+^ can form with bismuth oxide a discrete phase of the same symmetry. An appealing explanation is the concept of a clathrate- or cage-type structure, in which as postulated by Sillén [1] for Si_2_Bi_24_O_40_, central Si atoms are surrounded by spheres of Bi_12_O_20_ atoms. For the case of a central ion with valence different from 4, charge balance would be achieved through cation or oxygen adjustments.

Regarding the metastable b.c.c. phase, the monovalent ion Rb^+^ formed the phase on cooling in the high temperature x-ray experiments. The compositions containing NiO, MnO, and Nb_2_O_5_, which in the process of preparation were ground in alcohol, also formed the b.c.c. phase, metastably. These results would seem to support the conclusion that an impure b.c.c. phase of Bi_2_O_3_ might be formed (metastably) with most cations, under the proper conditions of composition, grinding, and heating schedules.

It is seen from figure 7 that with the exception of PbO the unit cell dimensions of the metastable phases are larger than those of the stable phases. The cell dimensions of the metastable impure phases, also, are less than those for the b.c.c. metastable phase of pure Bi_2_O_3_. Therefore, the compositions of the impure metastable phases cannot be that of pure Bi_2_O_3_. However, contrary to the case of the stable b.c.c. phases, no correlation exists between unit cell dimensions and ionic radius for the metastable b.c.c. phases. The x-ray diffraction patterns of the b.c.c. phases of the stable and metastable impurity forms are similar in *d* spacings and intensities to the pattern for pure Bi_2_O_3_. It is a reasonable assumption that the structures are similar.

To summarize (see fig. 7), the b.c.c. phase of pure Bi_2_O_3_ has the largest unit cell dimensions, and the addition of a foreign ion to Bi_2_O_3_ tends to decrease the dimensions. This decrease is least for the larger ions, which tend to form the metastable b.c.c. phase. The decrease in cell dimensions is greatest for the smaller ions, which tend to form the stable b.c.c. phase. Whereas the stable b.c.c. phases show correlation with ionic radius, the metastable phases do not. These findings are compatible with a cage-type structure in which a central cation, including Bi, is surrounded by a sphere of atoms of approximately Bi_12_O_20_ composition.

## 4. Summary

**Figure 8:**
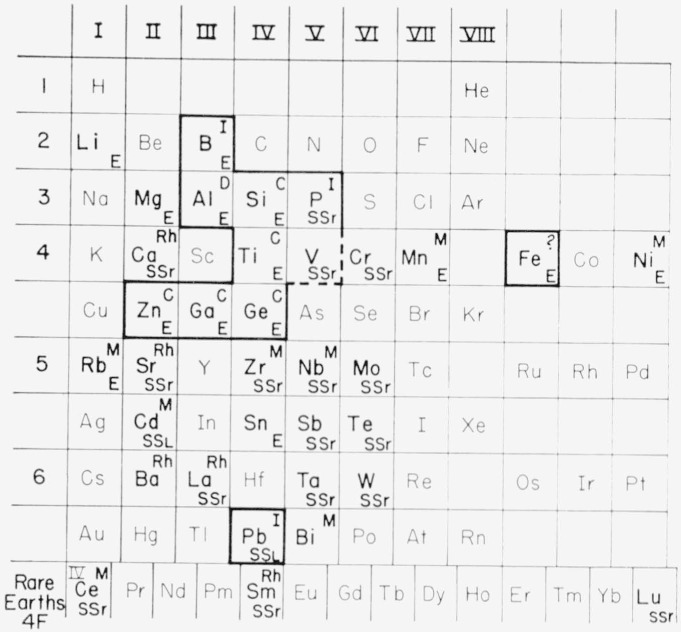
Summary of some phase equilibria data on the effect of oxide additions to *Bi_2_O_3_*. Elements shown in boldfaced type on the Periodic Table refer to oxide additions Elements enclosed in heavy outlines represent oxides which form stable body- centered cubic phases with bismuth oxide: C—congruently melting I—incongruently melting D—decomposes M—metastable body-centered cubic phase E—eutectic-type system SS_r_—solid solution of oxide in Bi_2_O_3_, with solidus and liquidus raised SS_L_—solid solution, with solidus and liquidus lowered Rh—rhombohedral solid solution phase [7, 8].

The important phase equilibria relationships for the bismuth-rich portions of the phase diagrams are shown schematically in figure 8. Elements in boldfaced type refer to the respective oxide mixtures studied. Elements enclosed in heavy outlines represent oxides which formed the stable b.c.c. phase with bismuth oxide. Composition of the b.c.c. phase was found to be variable for different systems, but most nearly approached the ideal 12Bi:1Me ratio for oxides of the tetravalent ions Si^4+^, Ti^4+^, and Ge^4+^. Designations in the upper right-hand corners of the boxes for the stable b.c.c. phases refer to the nature of melting, e.g., congruent, incongruent, or decomposition. Oxides of elements which formed the metastable b.c.c. phase are indicated by an *M* in the upper right-hand corner of the box, and those that formed the rhombohedral solid solution phase, by *Rh.* The nature of the liquidus curves is indicated by a designation in the lower right-hand corner of each box, as follows: *E*, simple eutectic; ss_r_, solid solution type with liquidus and solidus raised; ss_L_, solid solution type with liquidus and solidus lowered.

## Figures and Tables

**Table 1:** Unit cell demensions of rhombohedral phases formed in *Bi_2_O_3_* systems

Oxide addition	Starting composition	Final heat treatment	Additional phases present	Ionic radius of cation (Ahrens)	Unit cell dimensions				
					Hexagonal			Rhombohedral	
									
		*°C/hr*			*a*	*c*	*c/a*	*a*	*α*
					A	A		A	
CaO^a^	6Bi_2_O_3_:CaO	700°/107	None	0.99	3.94_1_	27.9_5_	7.09	9.59_0_	23°44′
SrO^a^,^b^	19Bi_2_O_3_:2SrO	699°/144	Mon Bi_2_O_3_ (moderate)	1.12	3.95_2_	28.0_9_	7.11	9.63_7_	23°40′
BaO^a^	6Bi_2_O_3_:BaO	700°/107	b.c.c. Bi_2_O_3_ (small)	1.34	3.97_2_	28.5_4_	7.18_5_	9.78_6_	23°26′
Sm_2_O_3_	12Bi_2_O_3_:Sm_2_O_3_	700°/107	tr?	1.00	3.95_0_	27.9_3_	7.07	9.58_5_	23°46′
La_2_O_3_	12Bi_2_O_3_: La_2_O_3_	700°/107	b.c.c. Bi_2_O_3_ (moderate)	1.14	3.97_5_	28.1_0_	7.07	9.64_4_	23°48′

a Rhombohedral phase described by Aurivillius [7].

b Rhombohedral phase described by Sillen and Aurivillius [8].

**Table 2:** Unit cell dimensions of body-centered cubic phases formed by small additions of oxides to Bi_2_O_3_

Stable phases						
Oxide addition	Starting composition	Final heat treatment	Additional phases present	Ionic radius of cation (Ahrens)	Levin and Roth	Aurivillius^a^ and Sillen [15]
						
		° *C/hr*		*A*	*A*	*A*^a^
ZnO	6Bi_2_O_3_:ZnO	700°/107	Trace ZnO	0.74	10.20_1_	
PbO	6Bi_2_O_3_:PbO	725°/1	None	1.20	10.25_8_	10.25^b^
B_2_O_3_	12Bi_2_O_3_:B_2_O_3_	600°/65	Trace Bi_2_O_3_+ trace 2:1	0.23	10.12_4_	
Al_2_O_3_	12Bi_2_O_3_: Al_2_O_3_	700°/107	Trace Bi_2_O_3_·2Al_2_O_3_	.51	10.16_9_	10.16
Ga_2_O_3_	12Bi_2_O_3_: Ga_2_O_3_	700°/107	Trace Bi_2_O_3_·2Ga_2_O_3_	.62	10.17_6_	
Fe_2_O_3_	19Bi_2_O_3_:Fe_2_O_3_^c^	700°/3	Trace BiFeO_3_	.64	10.18_3_	10.18
Tl_2_O_3_				.95		10.17
SiO_2_	6Bi_2_O_3_:SiO_2_	700°/107	Small amt. Bi_2_O_3_	.42	10.10_4_	10.10
GeO_2_	6Bi_2_O_3_: GeO_2_	700°/107	Trace GeO_2_	.53	10.14_3_	
TiO_2_	6Bi_2_O_3_:TiO_2_	700°/107	None	.68	10.17_6_	
P_2_O_5_	12Bi_2_O_3_:P_2_O_5_	700°/107	Trace?	.35	10.16_4_	

Metastable phases						

Rb_2_O	12Bi_2_O_3_: Rb_2_O	795°/0.1^d^	Trace Bi_2_O_3_	1.47	10.22	
NiO	Bi_2_O_3_:NiO^c^	785°/1	NiO	0.69	10.22_7_	
MnO	Bi_2_O_3_:MnO^c^	735°/1	Moderate amt. Bi_2_O_3_·2Mn_2_O_3_	.80	10.22_1_	
CdO	6Bi_2_O_3_:CdO	700°/107	Trace CdO	.97	10.22_5_	
Bi_2_O_3_	Bi_2_O_3_	780°/0.1^e^	Small amt. Mon Bi_2_O_3_	1.20^f^	10.26_8_	10.264^g^
ZrO_2_				0.79		10.23
CeO_2_				.94		10.22
V_2_O_5_	12Bi_2_O_3_: V_2_O_5_	700°/107	Small amt. Tet. Bi_2_O_3_ ss	.59	10.21_0_	
Nb_2_O_5_	6Bi_2_O_3_:Nb_2_O_5_^c^	700°/3	Moderate amt. cubic (C′ [17])	.69	10.19	
Nb_2_O_5_	24Bi_2_O_3_: Nb_2_O_5_ c	700°/3	Trace cubic (C′ [17])	.69	10.26_3_	

a Converted from kx units.

b Cation is tetravalent according to Aurivillius and Sillen [15].

c Starting materials ground together in alcohol.

d Sample cooled from liq. in high-temp., x-ray furnace.

e Slow-cooled in high-temp., x-ray furnace.

f Ahrens gives 0.96Å, which is low. See [4].

g Agrees with value obtained by Schumb & Rittner [2].

## Footnotes

1 Figures in brackets indicate the literature references at the end of this paper.
